# Association between central obesity indices and iron status indicators among Qatari adults

**DOI:** 10.1371/journal.pone.0250759

**Published:** 2021-04-29

**Authors:** Abdelhamid Kerkadi, Reem Mohsen Ali, Alaa A. H. Shehada, Eman Abdelnasser AbouHassanein, Joyce Moawad, Hiba Bawadi, Zumin Shi

**Affiliations:** Human Nutrition Department, College of Health Sciences, Qu-Health, Qatar University, Doha, Qatar; Shahjalal University of Science and Technology, BANGLADESH

## Abstract

Co-existence of iron deficiency and obesity in adults has been reported in many countries. However, little is known on the association between obesity and iron deficiency in Qatar. This study aimed to investigate the link between abdominal obesity indices and iron status among adults in Qatar. A random sample of 1000 healthy Qatari adults, aged 20–50 years, was obtained from Qatar Biobank study. Body weight, waist circumference, dual x-ray absorptiometry (DXA) parameters and iron status indicators were measured using standard techniques. Multiple regression analysis was used to examine the associations. The prevalence of iron deficiency and anaemia were 35.4 and 25%, respectively. Of the participants with a large waist circumference, 31.7% had anaemia. Ferritin significantly increased with the increase in the android fat to gynoid fat ratio and visceral fat in both genders. Serum iron and transferring saturation decreased significantly with the increase in waist circumference in women. In both genders, C-reactive protein increased with the increase in all obesity indices. Standardized values of waist circumference, android fat, gynoid fat ratio and visceral fat were significantly associated with log transformed ferritin in men and women. Waist circumference was inversely related to serum iron (β:-0.95, 95% CI:-1.50,-0.39) and transferrin saturation (β:-1.45, 95%CI:-2.46, -0.43) in women. In men, waist circumference was positively associated with haemoglobin level (β: 0.16, 95% CI:0.04, 0.29). Central obesity coexists with anaemia among the study population. Elevated central obesity indices were associated with an increase in ferritin concentration. The increased ferritin concentration may be attributed to the increase in inflammatory status as a result of an increase in c-reactive protein concentration associated with central obesity.

## 1. Introduction

Obesity and iron deficiency (ID) continue to be a worldwide public health problem [1]. In Qatar, the percentage of overweight (OW) and obese people is alarmingly high. Results of the WHO STEPwise survey indicated that 28.7 and 41.1% of the Qatari population are OW and obese, respectively [2]. The prevalence of central obesity (CO) is 48.4% (45.4% in men and 51.3% in women) [3]. Several epidemiological studies have documented a strong association between obesity indices and the development of cardiovascular disease, such as coronary heart disease and stroke [4–7]. A recent study revealed that 16% of the total population of Qatar have one or more of these four non-communicable diseases, namely cardiovascular disease, type 2 diabetes mellitus, cancer and chronic obstructive pulmonary diseases. Among the four non-communicable diseases, cardiovascular disease and type 2 diabetes mellitus are the most common [8]. In addition to the burden of obesity and non-communicable diseases, micronutrient deficiencies, e.g. iron and vitamin D, continue to be a public health concern in Qatar [9–11]. The WHO estimated that 28% of non-pregnant women are anaemic [11]. The co-occurrence of obesity and micronutrient deficiencies has been reported in many countries [12–18]. Data from population-based surveys indicated that, on average, 8.6% (range 1–18.6%) of women of reproductive age in seven countries were facing the double burden of obesity and anaemia [14]. A systematic review further indicated that 12.6% of households were suffering from the double burden of obesity and anaemia [12]. Another study of women from three countries that were undergoing the nutrition transition found an overlap between being OW and having anaemia [19].

Obesity is known as an accumulation of fat in the adipose tissue (subcutaneous and/or visceral tissue). Most of the studies that were conducted to elucidate the relationship between obesity and ID used body mass index (BMI) as an indicator of obesity. However, BMI is not considered to be a direct measure of adiposity. The effect of fat distribution, especially in CO, on iron status remains controversial [20–23]. It is well known that obesity affects iron metabolism by impairing the absorption of iron and decreasing the body’s ability to store it [19]. Different hypotheses have been suggested to elucidate the relationship between obesity indices and ID. One of the major hypotheses is related to the inflammatory process. The high rate of ID among people suffering from obesity may be due to the chronic inflammatory state induced by excess weight. Adipose tissue is an active endocrine organ that increases the secretion of inflammatory mediators in obese patients. Several proinflammatory mediators are released by the adipose tissue, including adiponectin, resistin, leptin, interleukin 6 (IL6), tumour necrosis factor (TNFα) and c-reactive protein (CRP) [1]. Stoffel et al. indicated that the release of IL6 by the visceral fat (VF) in patients with CO could strongly stimulate the synthesis of hepcidin [20]. A recent study found that obese people, especially those with abdominal obesity, have high serum hepcidin and ferritin levels and reduced iron availability owing to increased inflammatory factors [24]. The increased level of hepcidin negatively affects iron absorption and may lead to ID [25]. It should be noted that ferritin is also an acute-phase protein regulated by TNFα and IL6 and upregulated in inflammatory states [26]. Results of a systematic review indicated that obese subjects tended to have higher hemoglobin and ferritin concentrations and lower transferrin saturation compared to non-obese subjects [18]. However, other studies did report any association between obesity and iron status indicators [27–29]. According to knowledge based on the available literature, no studies have been conducted to evaluate the prevalence of ID in adults with CO in Qatar. Therefore, this study aims to examine the association between CO and iron status in men and women in Qatar.

## 2. Methods

### 2.1. Study population

This study is a population-based, cross-sectional survey of adults aged 20–50 years who are either Qatari or long-term residents of Qatar (≥15 years). This study was conducted within the framework of Qatar Biobank (QBB). It is the first national, Qatar, population-based prospective cohort study that includes the collection of biological samples with long-term storage of data and samples for future research [30]. In this study, 1000 samples from men and women aged 20–50 years were selected from the master database by the QBB management group. Subjects fulfilling the inclusion and exclusion criteria were selected using a simple random selection in which each person had the same chance of being selected. The sample size was selected because QBB provides 1000 free samples for research projects done by institutions in Qatar. Fourteen individuals with missing data were excluded. Therefore, 986 were included in the data analysis. Exclusion criteria were pregnant women, lactating women, chronic diseases (such as diabetes, hypertension, cancer and chronic kidney disease), endocrine disease (such as Cushing syndrome, heavy or irregular menses), a restrictive diet for weight loss, medical treatment with growth hormones or taking vitamin and mineral supplements.

The study was approved by the Institutional Review Board of QBB (EX-2018-RES-ACC-0118-0060) and was carried out in accordance with the Declaration of Helsinki, as revised in 2008. An informed written consent form was signed by each participant.

### 2.2. Obesity indicators, anthropometric and DXA derived parameters

Trained staff in QBB clinics measured the anthropometric indicators using standard methods. Body weight (kg) and height (cm) were measured with a calibrated scale and a wall-mounted stadiometer, respectively, in patients wearing light clothing and no shoes. Waist circumference (WC) was determined at the midpoint between the last rib and the top of the iliac crest using a stretch-resistant tape. Overall adiposity, total body fat (TBF), VF and regional fat distribution (trunk, android fat [AF] and gynoid fat [GF]) were measured using Lunar iDXA (SN 210520, GE Healthcare, USA). DXA-derived parameters were used to calculate the AF/GF ratio. CO was defined as WC ≥ 102 cm for men and WC ≥ 88 cm for women. OW was defined as BMI = 25–29.9 kg/m^2^ and obesity (OB) as BMI ≥ 30 kg/m^2^ [31]. Blood pressure was measured using the Omron 705 IT automated device (Omron Corporation, Kyoto, Japan). Diastolic blood pressure and systolic blood pressure were measured twice, with a third measurement being taken if there was a difference ≥5 mmHg between the two readings.

### 2.3. Measurement of biomarkers

Fasting blood samples were immediately collected from participants at the time of their visit. Blood samples were collected by a registered nurse at a QBB clinic. All biochemical assays were performed at the Medical Central laboratory of Hamad General Hospital in Doha, Qatar. Serum iron concentration was measured using the ferrozine method without deproteinization according to the manufacturer’s protocol provided with the Reagent (Cobas, Cat. No 05169291 190). In a weak acid buffer, iron is dissociated from the transferrin-iron complex. The released Fe^3+^ ions are reduced to Fe^2+^ions by ascorbate. Ferrous ions (Fe^2+^) form a coloured complex with Ferrozine. The color intensity is proportional to the iron concentration in the sample. The iron concentration was determined using the Cobas C701 module (Roche Diagnostic, Mannheim, Germany). Ferritin was determined by electrochemiluminescence immunoassay (ECLIA), based on the sandwich principle using the Reagent (Cobas, Cat. No 04491785190). The combination of ten μL of the sample with a biotinylated monoclonal ferritin-specific antibody, and a monoclonal ferritin- specific antibody labeled with a ruthenium complex- forms a sandwich complex. After the addition of streptavidin-coated microparticles, the complex becomes bound to the solid phase via the interaction of biotin and streptavidin. The reaction mixture is aspirated into the measuring cell, where the microparticles are magnetically captured onto the surface of the electrode. Unbound substances are then removed with ProCell/ProCell M. Application of a voltage to the electrode then induces chemiluminescent emission, measured by the Cobas 8000–1 modular platform (Roche Diagnostic, Mannheim, Germany). Transferrin was analysed by an immunoturbidimetric assay (Cobas, Cat. No 05588855 190). This procedure measures the increasing turbidity caused by the precipitate formed by adding a specific antibody to transferrin. The transferrin concentration was measured as a function of turbidity using the Cobas 8000 module (Roche Diagnostic, Mannheim, Germany). Total iron-binding capacity (TIBC) was assessed using the unsaturated iron-binding capacity (UIBC) method (Cobas, Cat. No 05975808 190). Excess of ferrous iron was added to the sample to saturate the transferrin. The remaining unbound iron ferrous ions were determined by the FerroZine method. The color intensity is directly proportional to the unbound excess iron concentration and indirectly to the unsaturated iron binding capacity. It was determined by measuring the increase in the absorbance using the Cobas 710 (Roche Diagnostic, Mannheim, Germany). TIBC represents the sum of iron serum and UIBC (TIBC = Iron serum + UIBC). The percentage of TS (%TS) was calculated by the formula %TS = serum iron ÷ TIBC. The Hb was assessed by cyanide-free sodium lauryl sulphate (SLS) method (Sysmex, Cat. No 90411414). The reagent lyses red blood cells and white blood cells in the sample. The chemical reaction begins by altering the globin and then oxidizing the haeme group. SLS’ hydrophilic groups can bind to the haeme group and form a stable, colored complex (SLS-Hb), and the concentration was measured using Sysmex XN Series (XN3100 and XN1000) (Kobe, Japan).

### 2.4. Covariates

The education level, age, smoking status and physical activity of the participants were obtained using a self-administered health and lifestyle questionnaire. Education was divided into three levels namely low education (up to secondary school), medium education (technical or professional school) and high education (university and above). Physical activity levels, expressed as metabolic equivalents (METs) in hours per week, were calculated based on the frequency and duration of the different types of physical activity. Only physical activity that was done during the patients’ leisure time was considered as physical activity in the calculation. Participants were divided in subgroups (tertiles) based on their METs. Participants were divided into groups based on their smoking status. These groups were current smokers, those with a history of smoking (ex-smokers) and non-smokers. Face-to-face interviews were done by a professional nurse to collect information on the patients’ health status, family history of disease and current medications.

### 2.5. Statistical analysis

Data were analysed using Stata 15.1. Results were presented as mean and standard deviations (SD) for continuous variables and percentages for categorical variables, to summarise the characteristics of the study population. The t test and chi-square test (χ^2^) were used to compare body adiposity and iron status parameters by gender, obesity indicators (central obesity, AF/GF ratio tertile, and VF tertile). WC, VF and AF/GF ratio were converted to a z-score. Serum ferritin was logarithmically transformed. Multiple regression was performed to assess the association between the independent factors, zWC, zAF/GF ratio and z-VF, and iron status indicators, log ferritin, Hb, iron, TS and TIBC. Values were adjusted for age, education level, smoking and leisure-time physical activity (METs, recoded as tertiles). Anaemia was defined as Hb < 12 g/100 ml for women and Hb < 13 g/100 ml for men. ID was defined as serum ferritin <15 μg/L [32]. Statistical significance was detected at p < 0.05.

## 3. Results

The characteristics of the samples by gender are given in Table 1. Of the sample, 32% were men. The mean age was 31.1 ± 7.3. Of the study population, 70% had achieved a bachelor’s degree or higher, 26.7% were either smokers or ex-smokers and 52.1% reported low leisure-time physical activity. There were significant differences in education level, smoking status and physical activity between genders. The prevalence of OW, OB and CO was 32.7%, 23.5% and 14.2%, respectively. We noted a significant difference between genders. The means of WC, Hb, TS, iron serum and ferritin were significantly higher in men than in women. 17.3% of the study population had higher CRP 24.7% and 31.9% of subjects had ID and anemia, respectively. The prevalence of anaemia and ID were significantly higher in women than in men.

**Table 1:** Sample characteristics by gender.

	Total	Male	Female	p-value
	N = 986	N = 316	N = 670	
Age (years)	31.1 (7.3)	30.6 (6.9)	31.4 (7.4)	0.099
BMI (kg/m^2^)	26.4 (5.6)	25.4 (5.0)	26.8 (5.8)	<0.001
BMI status N (%)				<0.001
Normal	432 (43.8)	154 (48.7)	278 (41.5)	
OW	322 (32.7)	118 (37.3)	204 (30.4)	
Obese	232 (23.5)	44 (13.9)	188 (28.1)	
WC (cm)	79.0 (71.0–87.0)	83.0 (75.0–90.0)	77.0 (69.0–84.0)	<0.001
Fat percentage	39.3 (31.6–45.1)	29.8 (23.2–34.1)	42.9 (38.6–47.0)	<0.001
Education N (%)				0.003
Low	62 (6.3)	11 (3.5)	51 (7.6)	
Medium	234 (23.8)	63 (20.0)	171 (25.6)	
High	687 (69.9)	241 (76.5)	446 (66.8)	
Smoking status N (%)				<0.001
Non smoker	723 (73.3)	106 (33.5)	617 (92.1)	
Smoker	136 (13.8)	123 (38.9)	13 (1.9)	
Ex-smoker	127 (12.9)	87 (27.5)	40 (6.0)	
LTPA N (%)				0.038
T1	514 (52.1)	148 (46.8)	366 (54.6)	
T2	145 (14.7)	57 (18.0)	88 (13.1)	
T3	327 (33.2)	111 (35.1)	216 (32.2)	
CRP N (%)				<0.001
<6 (mg/L)	771 (78.2)	263 (83.2)	508 (75.8)	
≥6 (mg/L)	171 (17.3)	33 (10.4)	138 (20.6)	
TS (%)	25.1 (13.4)	34.2 (12.7)	20.8 (11.4)	
Hb (g/dl)	13.0 (12.0–14.4)	14.9 (14.2–15.5)	12.4 (11.5–13.1)	<0.001
Serum iron (μmol/L)	14.0 (9.4–18.8)	17.4 (14.0–22.1)	12.0 (7.7–16.9)	<0.001
Log ferritin (μg/L)	3.2 (2.3–4.1)	4.5 (4.0–4.9)	2.6 (2.1–3.3)	<0.001
TIBC (μmol/L)	60.0 (54.0–67.0)	54.0 (49.0–59.0)	63.0 (57.5–71.0)	<0.001
ID N (%)	345 (35.4)	10 (3.2)	335 (50.5)	<0.001
Anaemia N (%)	244 (25.0)	12 (3.8)	232 (34.9)	<0.001
CO N (%)	140 (14.2)	24 (7.6)	116 (17.3)	<0.001

Data are presented as mean (SD) or median (IQR) for continuous measures and N (%) for categorical measures. Normal TS: transferrin saturation; TIBC: total iron binding capacity; CRP: C reactive protein; Normal: BMI <25 kg/m^2^, overweight (OW) = 25–29.9 kg/m^2^, obese ≥ 30 kg/m^2^; central obesity (CO) is defined as having a WC ≥ 102 cm for men and WC ≥ 88 cm for women; Education level: low is up to secondary school, medium is a professional qualification and above and high is a university degree and above; Anaemia: Hb < 12 g/100 ml for women and Hb < 13 g/100 ml for men; ID: iron deficiency defined as serum ferritin <15 μg/L; Leisure time physical activity tertiles: T1 = 0 METs, T2 = 0.5 METs and T3 = 52.5 METs.

Table 2 shows iron status grouped by WC categories and gender. Results indicated that participants with higher WC had significantly lower Hb, serum iron and TS compared to participants with normal WC. We noted a significant increase in serum CRP and in the prevalence of anemia with an increase in WC. The results indicated that women with a large WC had significantly lower serum iron (p = 0.005) and TS (p = 0.048). It was also observed that there was a significant (p < 0.001) increase in CRP with an increase in WC in both genders. In men, only log ferritin significantly differed between the WC groups (p = 0.007). There were no significant differences in the percentages of anaemia and ID between groups for either gender.

**Table 2:** Comparison of iron status indicators between people with normal and large WC.

	Total	Normal WC	High WC	p-value	
	N = 986	N = 846	N = 140		
Total				
Log ferritin (μg/L)	3.2 (1.2)	3.3 (1.2)	3.1 (1.2)	0.19
Hb (g/dl)	13.1 (1.8)	13.1 (1.7)	12.6 (1.8)	<0.001
Iron (μmol/L)	14.6 (6.8)	14.9 (6.8)	12.4 (6.2)	<0.001
TS (%)	25.1 (13.4)	25.7 (13.5)	21.2 (12.3)	<0.001
TIBC (μmol/L)	61.2 (9.7)	61.2 (9.7)	61.4 (9.6)	0.77
CRP (mg/L)	4.8 (5.1)	4.4 (4.7)	7.4 (6.4)	<0.001
Anemia N(%)	244 (25.0)	200 (23.9)	44 (31.7)	0.049
ID N (%)	345 (35.4)	294 (35.2)	51 (36.7)	0.73
Men	**N = 316**	**N = 292**	**N = 24**	
Log ferritin (μg/L)	4.4 (0.7)	4.4 (0.7)	4.8 (0.7)	0.007
Hb (g/dl)	14.9 (1.0)	14.9 (1.0)	14.9 (1.3)	0.73
Iron (μmol/L)	18.3 (6.2)	18.4 (6.2)	17.5 (6.1)	0.50
TS (%)	34.2 (12.7)	34.3 (12.8)	33.0 (12.4)	0.64
TIBC (μmol/L)	54.8 (7.1)	54.9 (7.2)	53.6 (6.0)	0.42
CRP (mg/L)	4.2 (5.4)	3.9 (5.3)	7.3 (5.3)	0.005
Anemia N(%)	12 (3.8)	10 (3.4)	2 (8.7)	0.21
ID N (%)	10 (3.2)	10 (3.5)	0 (0.0)	0.36
Women	**N = 670**	**N = 554**	**N = 116**	
Log ferritin (μg/L)	2.7 (0.9)	2.6 (0.9)	2.8 (1.0)	0.18
Hb (g/dl)	12.2 (1.3)	12.2 (1.3)	12.1 (1.5)	0.34
Iron (μmol/L)	12.8 (6.3)	13.1 (6.4)	11.3 (5.7)	0.007
TS (%)	20.8 (11.4)	21.2 (11.5)	18.8 (10.9)	0.045
TIBC (μmol/L)	64.2 (9.2)	64.5 (9.1)	63.0 (9.4)	0.11
CRP (mg/L)	5.1 (5.0)	4.6 (4.4)	7.4 (6.6)	<0.001
Anemia N (%)	232 (34.9)	190 (34.7)	42 (36.2)	0.75
ID N(%)	335 (50.5)	284 (51.8)	51 (44.0)	0.12

Data are presented as mean (SD) or median (IQR) for continuous measures and N (%) for categorical measures. Normal TS: transferrin saturation; TIBC: total iron binding capacity; CRP: C reactive protein; Normal: BMI <25 kg/m^2^, overweight (OW) = 25–29.9 kg/m^2^, obese ≥ 30 kg/m^2^; central obesity (CO) is defined as having a WC ≥ 102 cm for men and WC ≥ 88 cm for women; Education level: low is up to secondary school, medium is a professional qualification and above and high is a university degree and above; Anaemia: Hb < 12 g/100 ml for women and Hb < 13 g/100 ml for men; ID: iron deficiency defined as serum ferritin <15 μg/L; Leisure time physical activity tertiles: T1 = 0 METs, T2 = 0.5 METs and T3 = 52.5 METs.

Iron status indicators, according AF/GF ratio tertiles, are presented in Table 3. Results have shown a significant increase in log ferritin and CRP with an increase in AF/GF ratio in the total population, women and men. However, no significant difference for iron, Hb, TS and TIBC, according to AF/GF ratio were found for either gender. In women, a significant decrease in the percentage of ID was noted with an increase in AF/GF tertile (p = 0.028).

**Table 3:** Comparison of iron status indicators according to AF/GF ratio tertile.

	Total	T1	T2	T3	p-value
	N = 915	N = 306	N = 305	N = 304	
Log ferritin (μg/L)	3.3 (1.2)	3.1 (1.2)	3.3 (1.1)	3.4 (1.2)	0.006
Hb (g/dl)	13.1 (1.8)	13.0 (1.8)	13.1 (1.8)	13.1 (1.8)	0.86
Iron (μmol/L)	14.7 (6.9)	15.2 (7.3)	14.6 (6.5)	14.3 (6.7)	0.27
TS (%)	25.4 (13.6)	26.1 (14.4)	25.3 (12.9)	24.7 (13.3)	0.46
TIBC (μmol/L)	61.1 (9.7)	61.8 (10.0)	60.9 (9.4)	60.7 (9.8)	0.37
CRP (mg/L)	4.8 (5.2)	4.0 (4.4)	4.5 (4.3)	5.9 (6.4)	<0.001
Anemia N(%)	227 (25.0)	74 (24.5)	78 (25.8)	75 (24.8)	0.92
ID N(%)	316 (34.9)	116 (38.4)	109 (36.3)	91 (30.0)	0.079
Men	**N = 308**	**N = 103**	**N = 103**	**N = 102**	
Log ferritin (μg/L)	4.4 (0.8)	4.3 (0.8)	4.4 (0.6)	4.6 (0.8)	0.046
Hb (g/dl)	14.9 (1.0)	14.8 (1.0)	15.0 (1.0)	14.8 (1.1)	0.43
Iron (μmol/L)	18.4 (6.2)	19.3 (6.4)	18.1 (5.5)	17.8 (6.6)	0.17
TS (%)	34.4 (12.8)	35.9 (12.9)	34.2 (12.1)	33.1 (13.2)	0.29
TIBC (μmol/L)	54.7 (7.2)	55.0 (7.1)	54.3 (7.3)	54.8 (7.2)	0.78
CRP (mg/L)	4.2 (5.4)	3.3 (2.2)	3.9 (4.0)	5.4 (8.2)	0.023
Anemia N(%)	12 (3.9)	4 (3.9)	2 (2.0)	6 (5.9)	0.35
ID N(%)	10 (3.3)	4 (4.0)	2 (2.0)	4 (3.9)	0.65
Women	**N = 607**	**N = 203**	**N = 202**	**N = 202**	
Log ferritin (μg/L)	2.7 (0.9)	2.5 (0.9)	2.7 (0.9)	2.8 (0.9)	0.001
Hb (g/dl)	12.2 (1.3)	12.2 (1.3)	12.2 (1.2)	12.2 (1.5)	0.97
Iron (μmol/L)	12.8 (6.4)	13.1 (6.9)	12.9 (6.2)	12.5 (6.1)	0.66
TS (%)	20.8 (11.5)	21.2 (12.6)	20.8 (10.8)	20.5 (11.1)	0.81
TIBC (μmol/L)	64.4 (9.2)	65.2 (9.5)	64.3 (8.6)	63.8 (9.5)	0.30
CRP (mg/L)	5.1 (5.0)	4.4 (5.0)	4.8 (4.5)	6.2 (5.4)	0.001
Anemia N(%)	215 (35.8)	70 (35.0)	76 (38.0)	69 (34.3)	0.72
ID N(%)	306 (50.9)	112 (55.4)	107 (54.0)	87 (43.3)	0.028

Data are presented as mean (SD) or median (IQR) for continuous measures and N (%) for categorical measures. Normal TS: transferrin saturation; TIBC: total iron binding capacity; CRP: C reactive protein; Normal: BMI <25 kg/m^2^, overweight (OW) = 25–29.9 kg/m^2^, obese ≥ 30 kg/m^2^; central obesity (CO) is defined as having a WC ≥ 102 cm for men and WC ≥ 88 cm for women; Education level: low is up to secondary school, medium is a professional qualification and above and high is a university degree and above; Anaemia: Hb < 12 g/100 ml for women and Hb < 13 g/100 ml for men; ID: iron deficiency defined as serum ferritin <15 μg/L; Leisure time physical activity tertiles: T1 = 0 METs, T2 = 0.5 METs and T3 = 52.5 METs.

Table 4 presents the results of the comparison between iron status indicators and VF tertiles. Subjects categorised into the high VF tertile (T3) had higher log ferritin and CRP in the total population, men and women. Serum iron significantly decreased with an increase in VF in the whole population and in women. No significant differences in the prevalence of anemia or ID was found between the VF tertiles for either gender.

**Table 4:** Comparison of iron status indicators according to VF tertiles.

	Total	T1	T2	T3	p-value
	N = 913	N = 305	N = 305	N = 303	
Log ferritin (μg/L)	3.3 (1.2)	3.1 (1.2)	3.2 (1.2)	3.4 (1.2)	0.013
Hb (g/dl)	13.1 (1.8)	13.1 (1.7)	13.1 (1.8)	13.0 (1.9)	0.81
Iron (μmol/L)	14.7 (6.8)	15.6 (7.1)	14.6 (6.9)	13.9 (6.4)	0.011
TS (%)	25.4 (13.6)	26.8 (14.2)	25.3 (13.7)	24.1 (12.7)	0.058
TIBC (μmol/L)	61.1 (9.7)	61.8 (10.3)	60.7 (9.5)	60.7 (9.3)	0.28
CRP (mg/L)	4.8 (5.2)	3.6 (3.5)	4.3 (3.6)	6.5 (7.2)	<0.001
Anemia N(%)	226 (25.0)	67 (22.2)	75 (25.0)	84 (27.7)	0.29
ID N(%)	314 (34.8)	114 (37.5)	111 (37.2)	89 (29.6)	0.067
Men	**N = 308**	**N = 103**	**N = 103**	**N = 102**	
Log ferritin (μg/L)	4.4 (0.8)	4.3 (0.8)	4.4 (0.7)	4.6 (0.7)	0.010
Hb (g/dl)	14.9 (1.0)	14.8 (1.0)	15.0 (1.0)	14.8 (1.2)	0.25
Iron (μmol/L)	18.4 (6.2)	19.5 (6.2)	17.9 (6.5)	17.8 (5.7)	0.073
TS (%)	34.4 (12.8)	36.7 (12.9)	33.7 (13.6)	32.7 (11.6)	0.070
TIBC (μmol/L)	54.7 (7.2)	54.6 (7.9)	54.3 (6.8)	55.2 (6.8)	0.67
CRP (mg/L)	4.2 (5.4)	3.1 (2.0)	3.6 (2.7)	5.8 (8.7)	0.001
Anemia N(%)	12 (3.9)	3 (2.9)	2 (2.0)	7 (6.9)	0.16
ID N(%)	10 (3.3)	5 (4.9)	3 (3.0)	2 (2.0)	0.50
Women	**N = 605**	**N = 202**	**N = 202**	**N = 201**	
Log ferritin (μg/L)	2.7 (0.9)	2.5 (0.9)	2.6 (0.9)	2.8 (0.9)	0.010
Hb (g/dl)	12.2 (1.3)	12.2 (1.3)	12.2 (1.3)	12.1 (1.5)	0.85
Iron (μmol/L)	12.8 (6.4)	13.6 (6.7)	12.9 (6.5)	12.0 (5.8)	0.041
TS (%)	20.9 (11.5)	21.7 (12.0)	21.1 (11.7)	19.8 (10.9)	0.23
TIBC (μmol/L)	64.4 (9.2)	65.5 (9.4)	64.0 (9.0)	63.6 (9.1)	0.086
CRP (mg/L)	5.1 (5.0)	3.9 (4.0)	4.6 (3.9)	6.8 (6.3)	<0.001
Anemia N(%)	214 (35.7)	64 (32.2)	73 (36.7)	77 (38.3)	0.41
ID (%)	304 (50.8)	109 (54.2)	108 (54.3)	87 (43.7)	0.052

Data are presented as mean (SD) or median (IQR) for continuous measures and N (%) for categorical measures. Normal TS: transferrin saturation; TIBC: total iron binding capacity; CRP: C reactive protein; Normal: BMI <25 kg/m^2^, overweight (OW) = 25–29.9 kg/m^2^, obese ≥ 30 kg/m^2^; central obesity (CO) is defined as having a WC ≥ 102 cm for men and WC ≥ 88 cm for women; Education level: low is up to secondary school, medium is a professional qualification and above and high is a university degree and above; Anaemia: Hb < 12 g/100 ml for women and Hb < 13 g/100 ml for men; ID: iron deficiency defined as serum ferritin <15 μg/L; Leisure time physical activity tertiles: T1 = 0 METs, T2 = 0.5 METs and T3 = 52.5 METs.

**Fig 1:**
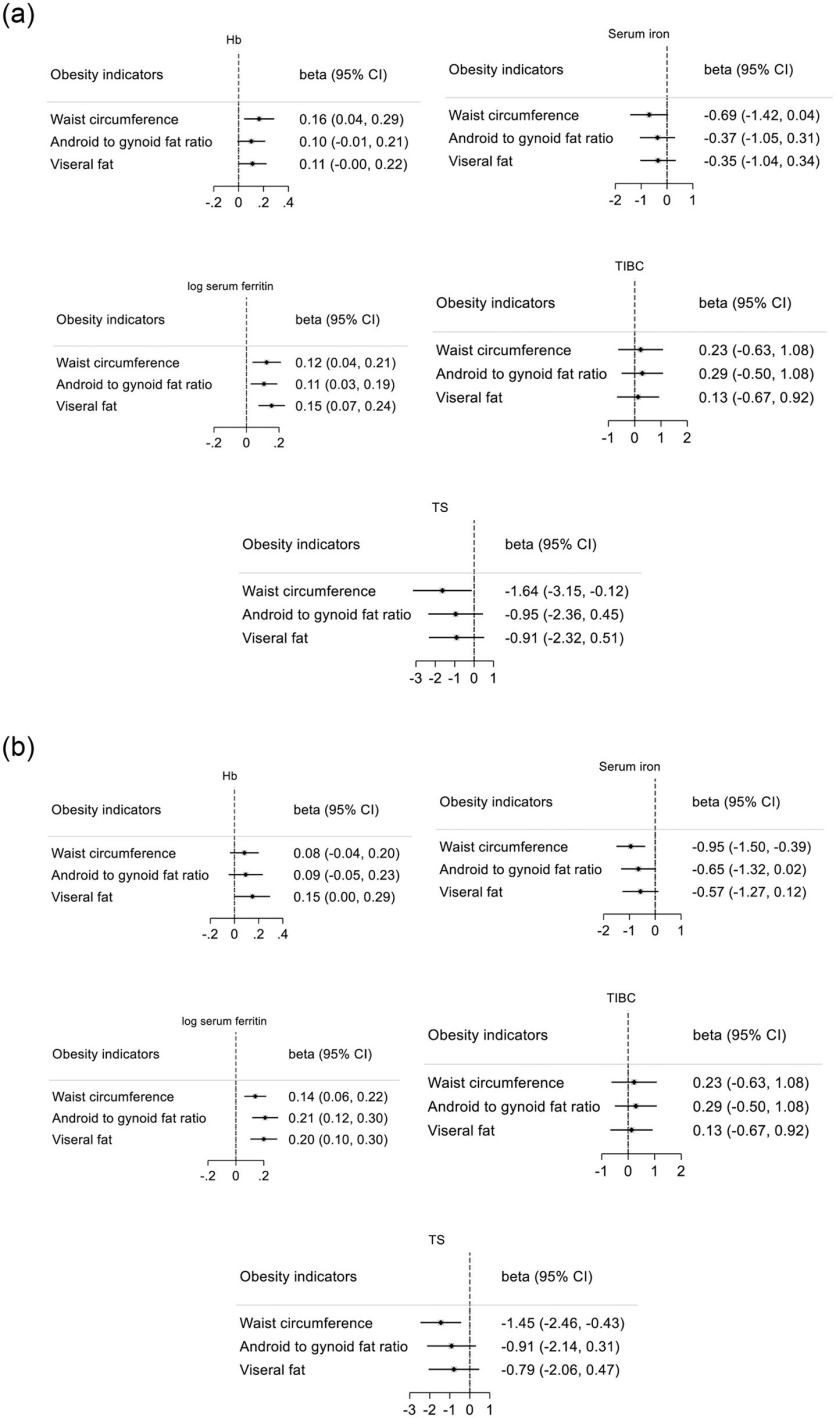
a: The correlation between the obesity indicators and the iron status indicators in men. b: The correlation between the obesity indicators and the iron status indicators in women.

Results of the multiple regression analyses are presented in Fig 1a and 1b. In men, z-WC was an inversely independent predictor of TS and a positive predictor of Hb. All abdominal obesity indices (z-WC, z-AF/GF and z-TS) were positive predictors of log ferritin. No correlation was found between any of the obesity indicators and TIBC or serum iron (Fig 1a).

In women, z-WC was an inversely independent predictor of serum iron and TS. Z-WC, z-AF/GF and z-VF were positive predictors of log ferritin. No relationship was found between Hb, TIBC or any of the abdominal obesity indices (Fig 1b).

## 4. Discussion

To our knowledge, the present study is the first to document the double burden of CO and ID in adults in Qatar. The results of the study indicate a high prevalence of CO (14.2%), anaemia (25%) and ID (35%) in the study population, with significant differences between the genders (34.9% in women and 3.4% in men). Similar results have been reported in KSA, Egypt and Tunisia with 40, 38.1 and 47.2% of women being anaemic, respectively [15, 33, 34]. However, the prevalence of anaemia in women in Lebanon and Morocco was 16 and 26.7%, respectively [15, 35]. Obesity and ID are the main nutritional problems in the world. Several researchers have reported the potential interaction between iron metabolism and excess body weight [36, 37]. The association between obesity and iron status remains controversial [16, 19, 38, 39].

This study revealed a high prevalence of ID and anaemia in adults with CO. The incidence of anaemia and ID were higher in women than in men. The incidence of anaemia in women with CO was higher in Qatar than in Tunisia and Morocco. Gartner et al. reported that 18.3 and 30.1% of women with a large WC had ID in Tunisia and Morocco, respectively. The same study also indicated the co-existence of anaemia and CO in women, 27.2% in Tunisia and 10.1% in Morocco [15]. It has also been reported that there is a significant difference in the prevalence of the double burden of CO and anaemia between the genders [40]. Qin et al. reported an inverse correlation between AO and anaemia in Chinese women [21]. Other studies have reported the co-existence of general obesity and anaemia [41–43]. In Chili, a study conducted among women of childbearing did not find any relationship between BMI and iron status [44].

Most studies have explored the relationship between iron status and obesity, determined by BMI, while limited studies have been done on the relationship between ID and anaemia using other obesity indicators such as WC, body fat and fat distribution. The results of this study indicated a significant decrease in iron and TS with an increase in WC in women, but not in men. This supports the results reported by Jordaan et al. who showed a significant increase in TS in women as WC increases [22]. In contrast, Choma et al. did not find any correlation, in men or women, between iron status or TS and WC [41]. In this study, women with a large WC did not have high serum ferritin. Other studies have showed similar results [22, 41].

However, Shim et al. reported a significant increase in ferritin, in both men and women, as WC increased [23]. Aderibigbe et al. found, in South Africa, that in women, ferritin increased with an increase in WC [45]. Results of a study conducted in China revealed a significant association between CO and high serum ferritin in women but not in men [46]. A prospective cohort study targeting Korean men showed an association between ferritin and WC [47]. Bergman et al. also reported a positive association between WC and ferritin in adults [48].

Recent studies have elucidated the relationship between fat distribution and iron status indicators [23, 45, 49–52]. Results of this study indicated a statistically significant increase (p < 0.05) in ferritin with an increase in android to gynoid fat ration for both sexes. A study conducted among healthy women using AF/TBF as an indicator of CO revealed a statistically significant increase in CRP and TIBC and a decrease in TS with an increase in AF/TBF. There were no associations between AF/TBF and other iron status indicators (ferritin, Hb, and serum iron) [20]. Results of a South Korean cross-sectional study demonstrated significantly positive correlations between serum ferritin and TBF in men and women. They also reported an increase in ferritin with an increase in trunk fat mass in both genders [23]. Another study, based on different fat distribution indicators, showed that ferritin was positively correlated with TBF and trunk fat mass and inversely correlated with leg fat mass in both genders [51]. Another study, conducted among young females, reported a statistically significant inverse correlation between body fat percentage and ferritin (r = −0.711, p = 0.029) [53]. However, Cheng et al. did not find any association between fat distribution (DXA variables) and iron status in Australian women [49]. This study found an increase in CRP with an increase in all CO indices for both men and women. Similar results have been reported in other studies [20, 49, 54]. The relationship between VS and iron status have not been extensively studied. We noted that participants (men and women) classified in the 3^rd^ tertile of VS had a higher level of ferritin when compared with those in the lowest tertile. This is consistent with the study of Iwasaski et al., who found a significant positive correlation between ferritin and VS (r = 0.254, p < 0.0001) [55]. In contrast to our findings, Cempaka et al. showed a weak correlation between VS and ferritin. This association disappeared after adjusting for age, gender and BMI [52].

The results of linear regression showed slight differences between the genders. In women, serum iron and TS were inversely associated with zWC, while in men, zWC was only positively associated with Hb. There was no gender–zWC interaction in relation to Hb. Log ferritin was positively related to all obesity indices (zWC, zAF/GF and zVF) in both genders. In contrast to our study, Choma et al. showed a positive relationship between WC and ferritin only in women. They also reported a positive and a negative relationship between WC and Hb for women and men, respectively [41]. In line with our study, Sabrina et al. revealed that the highest iron to ferritin ratio tertile, which reflects iron bioavailability, was associated with a decrease of 1.21 units in the VF mass in healthy women [50]. Several studies have shown that high ferritin is a risk factor for VF and metabolic disorders [56–58].

Different mechanisms have been proposed to elucidate the relationship between obesity and ID, including an iron-poor diet and an increased iron requirement due to an increase in blood volume, body fat and low-grade inflammation [59]. Recent studies reported that iron intake and its bioavailability were similar for obese and non-obese participants [44, 60, 61]. Among the three mechanisms, the systemic low-grade inflammation characterising obesity has received particular attention [29, 38, 62, 63]. The decrease in circulating iron observed in obese individuals has been linked to an increase in hepcidin expression due to chronic low-grade inflammation [63]. The activation of hepcidin can lead to a decrease in iron absorption and macrophage iron release through its downregulation [61]. An elevated concentration of hepcidin was observed among obese populations, despite the presence of hypoferremia [62–64]. In this study, we noted an increase in CRP with an increase in WC, AF/GF and VS. This is consistent with other studies that reported an increase in CRP in response to an increasing in obesity indices [20, 28, 41, 65]. This study indicated an increase in ferritin with an increase in AF/GF and VS. A higher ferritin should reflect an adequate store to support haematopoiesis. Iron is stored in the body as ferritin, an acute-phase reactant, which increases during inflammation [59, 63]. The higher ferritin concentration observed in participants with CO can be due to the chronic inflammation associated with obesity [26]. Ferritin is a biomarker of iron status and is widely used as an indicator of ID. Several studies have demonstrated the inaccuracy of serum ferritin concentrations as a measure iron stores under inflammatory conditions because it can underestimate the prevalence of ID [66–69]. Therefore, it was recommended to adjust the level of ferritin concentration for inflammation to correctly estimate the prevalence of ID in the population. Several studies have reported an underestimation of the prevalence of ID when using non-adjusted ferritin concentration [66–69]. In the context of the obesity burden in Qatar, it is important to assess the iron status and include the measurement of inflammatory biomarkers (CRP or high-sensitivity CRP). It is also highly recommended to adjust ferritin for inflammation to correctly estimate the prevalence of ID in individuals with excess weight. Future research may be required to see the effect of dietary habits and body composition on iron metabolism and the development of other non-communicable diseases.

This study has some limitations. First, in this study, we recognise the lack of sensitive inflammatory biomarker measures, which would have been useful in characterising the relationship between obesity and iron status. We used CRP as an indicator of possible inflammation. This study did not assess thalassaemia, a common disease in the region. Another limitation is due to the cross-sectional design of the study and therefore, no conclusions regarding the relationship between obesity and iron status could be drawn. The strengths of the study include the sample size, the body composition parameters gathered using DXA and the iron status variables.

## 5. Conclusion

Central obesity coexists with anaemia among the study population. Waist circumference was inversely related to serum iron and transferrin saturation in women. Elevated central obesity indices were associated with an increase in ferritin concentration. The increase in the ferritin concentration may be attributed to the high inflammatory status, reflected by an increase in CRP concentration with CO.

## Supporting information

S1 Checklist (DOC)
